# Surgical management of Crohn’s disease: a state of the art review

**DOI:** 10.1007/s00384-021-03857-2

**Published:** 2021-02-02

**Authors:** Elise Maria Meima - van Praag, Christianne Johanna Buskens, Roel Hompes, Wilhelmus Adrianus Bemelman

**Affiliations:** Department of Surgery, Amsterdam Gastroenterology and Metabolism, Amsterdam UMC, University of Amsterdam, Meibergdreef 9, 1105 AZ Amsterdam, the Netherlands

**Keywords:** Surgery, Inflammatory bowel disease, Crohn’s disease, Gastrointestinal tract

## Abstract

**Purpose:**

The aim of this review was to examine current surgical treatments in patients with Crohn’s disease (CD) and to discuss currently popular research questions.

**Methods:**

A literature search of MEDLINE (PubMed) was conducted using the following search terms: ‘Surgery’ and ‘Crohn’. Different current surgical treatment strategies are discussed based on disease location.

**Results:**

Several surgical options are possible in medically refractory or complex Crohn’s disease as a last resort therapy. Recent evidence indicated that surgery could also be a good alternative in terms of effectiveness, quality of life and costs as first-line therapy if biologicals are considered, e.g. ileocolic resection for limited disease, or as part of combination therapy with biologicals, e.g. surgery aiming at closure of select perianal fistula in combination with biologicals.

The role of the mesentery in ileocolic disease and Crohn’s proctitis is an important surgical dilemma. In proctectomy, evidence is directing at removing the mesentery, and in ileocolic disease, it is still under investigation. Other surgical dilemmas are the role of the Kono-S anastomosis as a preventive measure for recurrent Crohn’s disease and the importance of (non)conventional stricturoplasties.

**Conclusion:**

Surgical management of Crohn’s disease remains challenging and is dependent on disease location and severity. Indication and timing of surgery should always be discussed in a multidisciplinary team. It seems that early surgery is gradually going to play a more important role in the multidisciplinary management of Crohn’s disease rather than being a last resort therapy.

## Introduction

Crohn’s disease is a chronic, granulomatous, inflammatory bowel disease which can affect the entire gastrointestinal tract and extraintestinal organs. Patients typically present with transmural, penetrating disease of the terminal ileum or colon. Crohn’s disease has the highest incidence and prevalence rates in western countries, with a peak incidence in adolescence and young adulthood [1]. Over the past decades, incidence and prevalence rates have been increasing most in newly industrialised countries [2–4]. This suggests that the still unclear aetiology is related to industrialisation and (western) lifestyle. Since Crohn’s disease is known for its intermittent and relapsing course and the extensive impact on patient’s quality of life, many different therapies have been studied. Usually, medical therapy is started as first line of treatment, whereas surgery was considered a last treatment resort when medical therapy had failed. Over the past decades, it became apparent that earlier surgery can be applied for certain disease variants and in patients with severe disease, especially now that surgical procedures are becoming more minimally invasive. Extensive small bowel resections resulting in short bowel syndrome, and permanent stomata should of course be avoided. Nowadays, around 3 out of 4 Crohn’s patients will undergo surgery during the course of their lives [5]. Especially patients with small bowel disease, perianal fistulas or diagnosed between the age of 45 to 59 years appear to have an increased risk of surgery [5]. Up to date, multiple surgical techniques have been reported for the various Crohn’s disease locations and disease behaviour.

According to the Montreal classification (based on the formerly used Vienna classification), Crohn’s disease location can be divided into ileal (L1), colonic (L2), ileocolonic (L3) and isolated (L4) upper disease (which can also be added to the first three when concomitant), and behaviour can be divided into non-stricturing and non-penetrating (B1), stricturing (B2) and penetrating (B3) types with or without perianal disease [6, 7]. Both disease location and behaviour are most important to evaluate when determining appropriate treatment strategies. Fistulas for example often arise from abscesses caused by perforating disease activity, although abscesses can also arise from an existing fistula if the drainage is blocked. Asymptomatic fistulas are usually not surgically treated, whereas enterovaginal and enterovesical fistulas are always treated surgically, and symptomatic perianal fistulas require seton drainage before initiating (or optimising) medical therapy or surgery aiming at closure as well.

In this state-of-the-art review, we aim to report beyond what has been published in the ECCO guidelines on the surgical management of Crohn’s disease [8] and will address current research questions relevant for the surgical management. A literature search was therefore performed in MEDLINE (PubMed) using the following search terms: ‘Surgery’ and ‘Crohn’. Relevant articles were reviewed for current treatment strategies. Different current surgical treatment strategies and considerations are discussed based on disease location, from top to bottom.

## Upper gastrointestinal Crohn’s disease

Crohn’s disease of the upper gastrointestinal tract exhibits itself in the oesophagus, stomach, duodenum and jejunum and can consist of, e.g. strictures, erosions/ulcerations, fistulas and a bamboo joint–like appearance in the stomach [9]. Although knowledge about Crohn’s disease has increased, and involvement of the upper gastrointestinal tract is a known predictor of recurrence and complications, data on treatment of upper gastrointestinal Crohn’s disease is still scarce [10]. Lesions in the upper gastrointestinal tract have been reported with large variation between 17 and 75%, unrelated to the amount of symptoms [11], with a marked increase over the past decades. Involvement of the upper gastrointestinal tract is reported in around 13% of patients in a recent multicentre cohort study by Greuter et al. [12] and occurrence in 41% according to Horjus Talabur Horje et al. [13].

Oesophageal Crohn’s disease has an estimated incidence of up to 6.5% in paediatric patients and ranging from 0.3 to 1.8% in adults [14–16]. However, it is probable that the true incidence rates are much higher due to infrequent diagnostics (endoscopy) of the upper gastrointestinal tract in asymptomatic Crohn’s patients [17, 18]. The mid and distal oesophagus are the most common sites for Crohn’s lesions such as ulcers, erosions and strictures [17]. Surgery is rarely indicated for such lesions, as usually endoscopic dilatation is sufficient for the strictures, but sometimes segmental resection is performed. Extraintestinal manifestations including pyoderma gangrenosum, spondylarthropathy and uveitis often accompany oesophageal Crohn’s disease.

Gastroduodenal Crohn’s disease is also quite rare and occurs in around 0.5 to 4% of Crohn’s disease patients [19]. Currently, there is still no consensus on the treatment of upper gastrointestinal Crohn’s disease available. Surgery should be considered in medically refractory patients as dysplasia and cancer can arise in persistent upper gastrointestinal strictures, mainly located in the duodenum [20]. Patients refractory to first-line medical treatment or with complications (stricture, fistula and abscess) usually undergo surgery. Also massive or persistent haemorrhage and gastric outlet or duodenal obstruction are known indications for surgery.

Stricturoplasties are mostly used for short strictures, where the term ‘short’ is considered less than 10 cm. Gastric strictures in the antrum and pylorus are generally managed by an antrectomy with Roux-en-Y bypass [21]; however, also laparoscopic bypass surgery is commonly used, with gastrojejunostomy.

## Ileal and ileocolonic Crohn’s disease

**Fig. 1:**
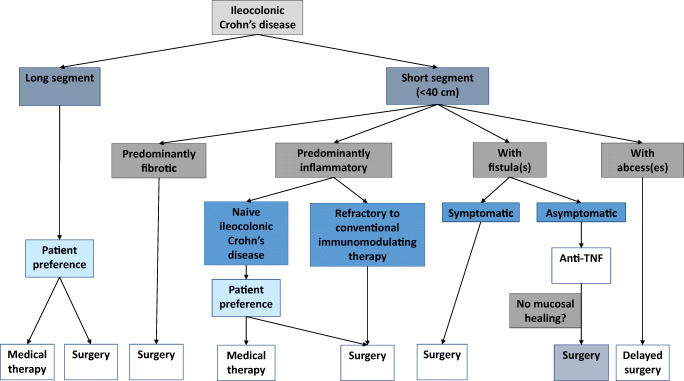
Flow chart for Crohn’s ileocolic treatment strategy

Consensus guidelines recognise the importance of surgery in complex ileocolonic disease with, e.g. abscesses, obstruction or sepsis. Currently, a staging tool is being developed which may enhance surgical decision-making for ileocolonic Crohn’s disease [22]. The surgical classification distinguishes (1) predominantly inflammatory ileal stricture, (2) predominantly fibrotic ileal stricture, (3) penetrating disease evident by fistulating disease (including enterovesicle/entero-vaginal/entero-cutaneous/multiple fistulae), and (4) perforating disease evident by intra-abdominal abscess or collection [22]. The staging tool is being validated using magnetic resonance enterography and computer tomographic enterography, and can help counsel patients of the incidence of for example concomitant surgery or stomata formation [22]. Indication for surgery, timing, type of approach and morbidity of the procedures are different for the various groups. A flow chart for treatment strategy of Crohn’s ileocolic disease is provided in Fig. 1.

### Predominantly inflammatory ileal stricture

Inflammatory Crohn’s strictures can be treated with medical therapy and surgery. Since the patients are primary diagnosed and treated by physicians, medical therapy is usually started and surgery is traditionally reserved for the patients not responding properly to the medical therapy.

This approach has been challenged by the results of the L!RIC study [23–25]. In this study, patients with uncomplicated Crohn’s terminal ileitis not responding to conventional medical therapy were randomised to have either anti-TNF or laparoscopic ileocolic resection. At 1 year after surgery, quality of life of the surgical patients was at least as good as of the anti-TNF patients if measured with the Inflammatory Bowel Disease Questionnaire (IBDQ), and better on the Short Form-36 (SF-36) [23]. Costs were significantly less for surgery [25]. At 5-year follow-up, none of the surgical patients required re-resection and only one quarter required a biological and almost half of these patients did not require any Crohn’s-related drugs [24]. In the anti-TNF group, half of the patients needed surgery and the other half was still taking a biological [24].

Laparoscopic ileocaecal resection can be performed with both single- and multiport laparoscopy. In single-port laparoscopy, the entire procedure can be facilitated through one extraction site, which not only improves cosmetic outcomes but is also associated with less need of postoperative pain medication [26]. Predictors of early postoperative recurrence after ileocaecal resection include smoking, previous intestinal surgery, penetrating disease, granulomas in resection and myenteric plexitis [21]. Dreaded complications after resection included septic complications, such as anastomotic leakage. According to Resegotti et al. [27], incidence rates of postoperative anastomotic leakage after elective ileocaecal or ileocolonic resection with ileocolonic anastomosis in Crohn’s patients range from 2% in patients with a stapled side-to-side anastomosis to 14% in patients with a handsewn end-to-end anastomosis. In case of recurrent disease, redo-surgery may be required. The extent of redo-resection should again be as minimal as possible, to reduce the imminent risk of intestinal failure. The cumulative risk of intestinal failure 20 years after the primary surgery is 8.5% [28]. It must be stressed that intestinal failure is rarely caused by the extent of the Crohn’s disease, and is mostly the result of inadvertent resection of damaged but healthy bowel during re-operative surgery for complications.

### Predominantly fibrotic ileal stricture

**Fig. 2:**
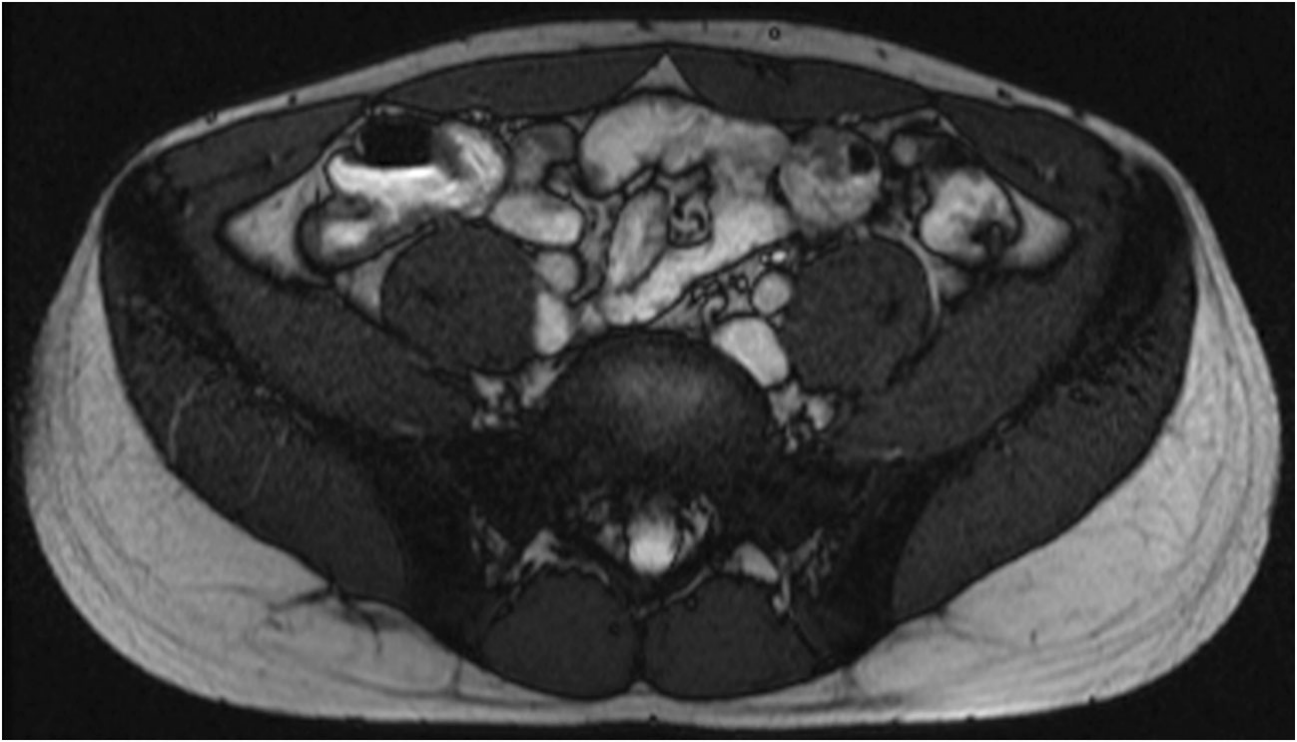
Markedly thickened (neo)terminal ileum with stenosis. Coronal T2-weighted post-contrast image of a patient with status after ileocaecal resection. MRI shows concentric wall thickening at the neoterminal ileum over a length of about 8 cm

If the Crohn’s stricture is predominantly fibrotic with little or absent enhancement on magnetic resonance imaging (MRI), the likelihood that medical therapy will benefit the patient is low. Particularly, if there is a prestenotic dilatation or the patient has obstructive symptoms, there is a clear indication for surgery (Fig. 2). In the presence of stenosis, surgery is often performed when endoscopic (balloon dilatation) and medical treatment failed or are deemed suboptimal. Surgery for (predominantly fibrotic) strictures usually entails stricturoplasty or segmental intestinal resection [29]. Many different types of stricturoplasties exist. The appropriate type of stricturoplasty is based on the length of the stricturised bowel and is combined with a segmental resection when necessary. The most commonly performed stricturoplasty is the Heineke–Mikulicz technique, where a longitudinal incision is made along the narrow section of the stricture and is transversely closed [30]. One of the main advantages of stricturoplasty is that the bowel is largely preserved, which is especially valuable in patients who have previously undergone significant bowel resection (> 100 cm). If needed, often, multiple stricturoplasties can be performed.

Short strictures can be managed by conventional stricturoplasties such as Heineke Mikulicz (< 10 cm) or Finney (10–25 cm). For long stenotic segments or multiple, close strictures, non-conventional stricturoplasties can be applied, e.g. the isoperistaltic side-to-side (or Michelassi) stricturoplasty can be used for segments up to 68 cm [21]. In the Michelassi stricturoplasty, the affected loop is divided halfway, the segments are placed side to side, and the segments are sutured together after a long incision between both loops [31]. Multiple fibrotic stricturoplasties are performed when technically feasible; otherwise, a single resection is performed when the patient has sufficient bowel length left.

According to Campbell et al. [32], the complication rates between the conventional (e.g. the Heineke–Mikulicz) and non-conventional (e.g. the isoperistaltic side-to-side) stricturoplasties including small bowel obstruction, sepsis, re-operation, bleeding, recurrence, carcinoma and mortality are similar. Contraindications for stricturoplasty include mucosal disease with active bleeding, fistulising disease, carcinoma or a phlegmon in the bowel wall [33].

### Terminal ileitis with fistulising disease

Entero-enteric fistulas, including enterocutaneous, enterosigmoid, enterocolonic and enterovesical fistulas, are reported in up to 30% of patients [34]. The indication for surgery is quite obvious if the patient is symptomatic, e.g. recurrent urinary tract infections, vaginal discharge or obstructive symptoms.

Entero-enteric fistulas are mostly asymptomatic and are only treated in the presence of a coexisting symptomatic stenosis. Even in the asymptomatic patients with fistulising disease not responding to medical management, surgery should be considered at an early stage, because smouldering disease could lead on the long run to large inflammatory masses incorporating a lot of otherwise healthy small and large bowel [35].

While managing enteral fistulas, resectional surgery should focus on the diseased organ. The target organ receiving the fistula must be spared by only excising the fistula tract and closing the opening. Proper preoperative imaging with colonoscopy and MRI is therefore important to rule out Crohn’s disease in the target organ.

### Perforating ileitis with intra-abdominal abscesses

Perforating enteral disease in Crohn’s disease can manifest with intra-abdominal abscesses. Small abscesses can be treated with antibiotics, but larger abscesses (>3 cm) require percutaneous drainage combined with medical therapy and when necessary followed by delayed resection. Delayed instead of instant resection should be pursued since this enables patients to improve their condition and is associated with less postoperative septic complications, a lower stomata rate, higher rate of laparoscopic surgery and a more limited resection [36]. Patients mostly develop these abscesses while on a biological indicating that there is little room for escalating therapy. Other factors supporting the indication for surgery are presence of a stenosis and small segment of involved bowel. The abscess must be drained, enteral or parenteral feeding must be started, antibiotics prescribed and anti-IBD medication stopped. It is advised to leave the drain in situ reducing the risk of a recurrent abscess. After 2 weeks, a laparoscopic resection can be attempted with risk of conversion. Whether the procedure must be staged depends on the condition of the patient and the surgeon’s preference. When it is decided to go for a staged procedure, the cross-stapled colon should be sutured to the abdominal wall close to the ileostomy, enabling stoma closure via a local procedure at a later stage.

## Surgical dilemmas of ileal and ileocolonic Crohn’s disease

### Stricturoplasty or resection

In the absence of fistulising disease, cancer or inflammatory mass, stricturoplasty is an alternative for resection. Long-term results indicate similar recurrences of resection as compared to stricturoplasty. It is obvious that short strictures can be managed by conventional stricturoplasties, e.g. Heineke Mikulicz (< 10 cm) or Finney (10–25 cm). Longer strictures require non-conventional complex stricturoplasties, e.g. Michelassi or Poggioli. The latter requires sufficient expertise which is only available in very few centres. If the sole purpose of stricturoplasties is to avoid small bowel loss, then one should realise that short bowel is rarely caused by the extent of the Crohn’s disease but rather by complication surgery where healthy bowel is unnecessarily sacrificed [37–39]. As reported by Peyrin-Biroulet et al. [40], the median length of small bowel resected after three resections was only 36 cm. So, with a median length of 3–5 m small bowel, resectional surgery even in extensive disease will rarely cause short bowel disease. The most important indication for the non-conventional stricturoplasties is extensive recurrent disease after prior resectional surgery.

### Mesenterectomy or close bowel dissection

**Fig. 3:**
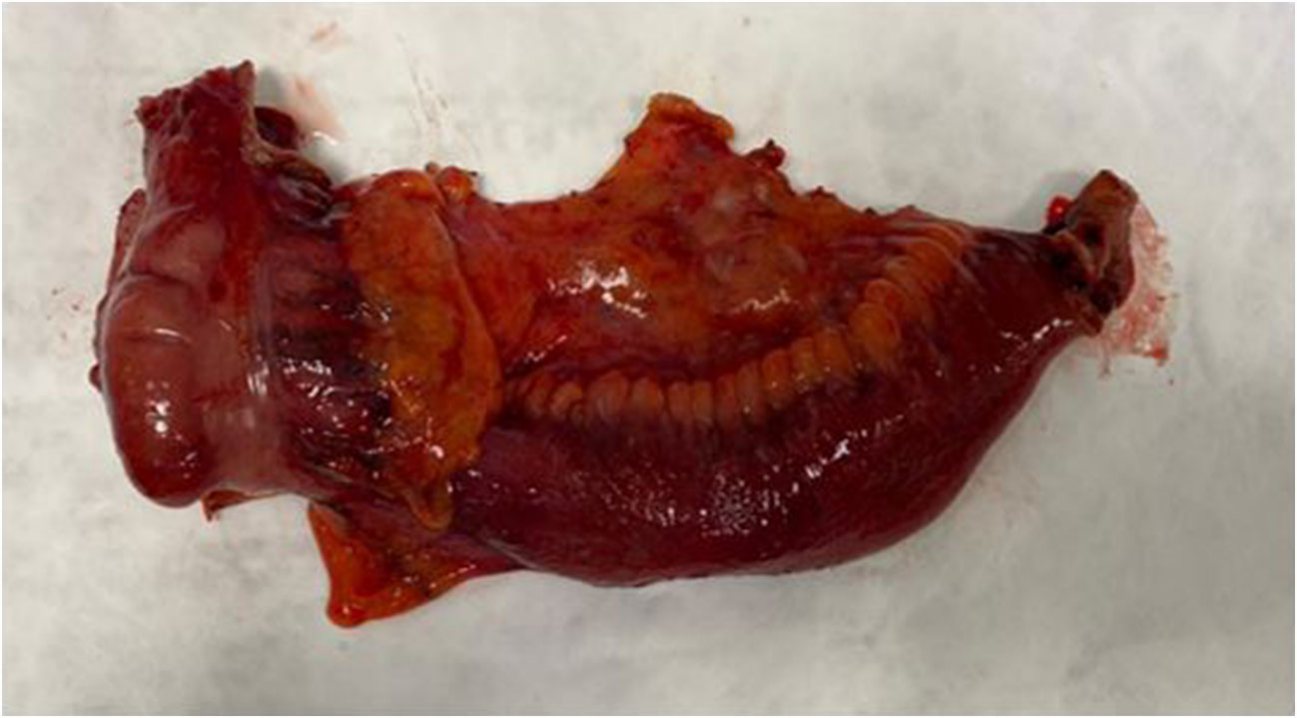
Extended mesenterectomy

**Fig. 4:**
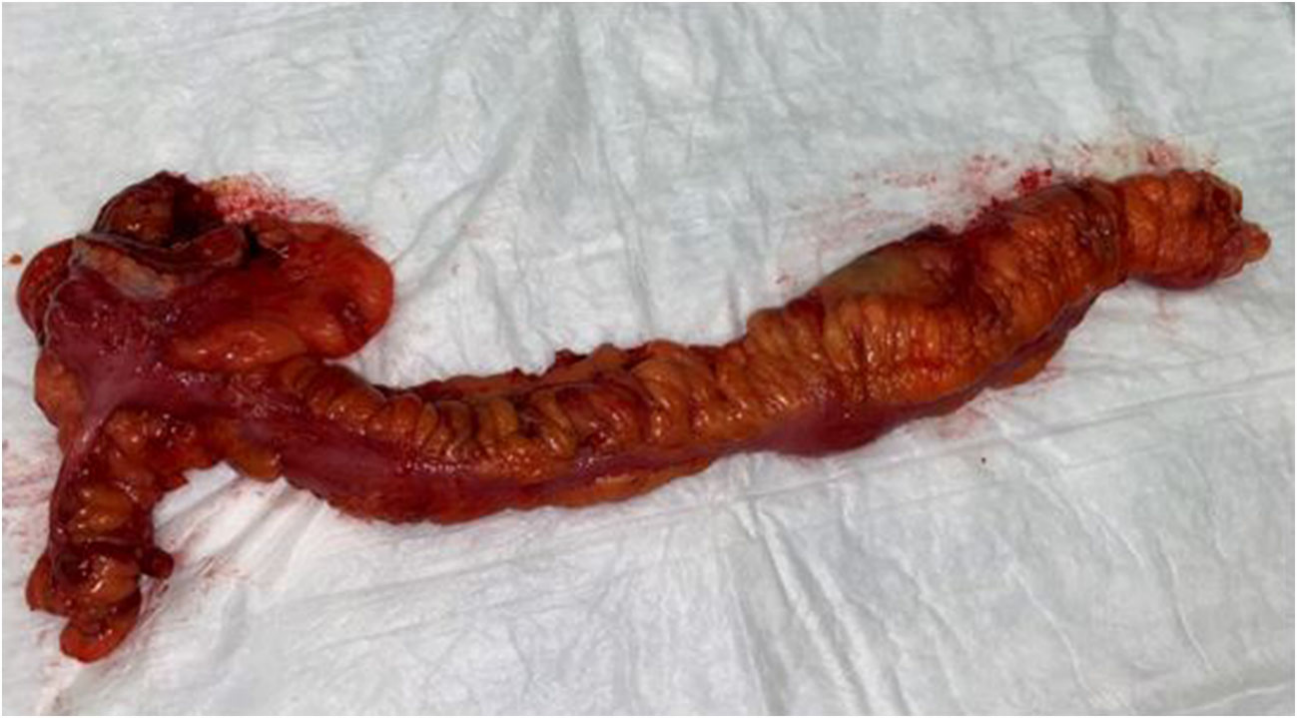
Close bowel ileocaecal resection

Recently, the mesentery is being rediscovered as a ‘separate organ’ which contains a lot of inflammatory cells and nerves. Up to date, it remains unclear what the role of the mesentery in Crohn’s disease is. Different opinions circulate on whether the mesentery is the driver of the disease or that what happens in the bowel is the cause of the changes in the mesentery. During both a proctectomy and ileocolonic resection, one should decide whether or not to take the mesentery too; the procedures can be performed either via total mesorectal excision (TME) or close bowel resection (Figs. 3 and 4). A typical expression around a Crohn’s disease lesion is creeping fat which locates primarily around the small bowel, usually the ileum, and expands and wraps itself around the inflammation. This usually occurs around stricturing or fibrotic lesions where according to Ha et al. [41], bacteria, including Clostridium innocuum, can translocate into the surrounding fat. Translocation can occur due to the inflammation, the stasis before a stricture and perhaps because of intraluminal hypertensia right before the stricture. This triggers a response from the M2 regulating macrophages which initiate massive fat production and fibrosis.

Another consideration is that the mesentery drives the Crohn’s disease of the bowel. In support of this, Coffey et al. [42] showed significantly lower recurrence rates after ileocolonic resection which included mesenterectomy compared to conventional, close bowel ileocolonic resection (2.9% versus 40%). However, the study compares a recent (operated after August 2010) with an earlier cohort (operated between January 2004 and April 2010), where the earlier cohort probably was not managed according to modern concepts including routine endoscopy after 6 months, faecal calprotectin and early initiation of therapy in case of endoscopic recurrence. Recent 5-year surgical recurrence rates as published by Stevens et al. [24] are similar or better than the mesenterectomy group of Coffey et al. [42] and the Kono-S anastomosis [43]. This suggests a rather large role of time and treatment effects, than an effect of the technique itself.

### Anastomotic techniques

The role of the mesentery is also relevant when deciding which type of stricturoplasty and anastomosis to apply. Since anastomotic lesions often recur on the side of the mesentery a new, end-to-end, hand-sewn ileocolonic anastomosis, the Kono-S was developed to prevent these anastomotic recurrences [44]. This basically side-to-side hand-sewn anastomosis is supported by the two stapled transection ends functioning as a supporting bridge which should avoid narrowing of the anastomosis. A recent randomised controlled trial compared the Kono-S to the conventional, stapled ileocolonic side-to-side anastomosis and showed that a lower endoscopic recurrence (Rutgeerts score ≥ i2) was reported 6 months after the Kono-S (22% versus 63%) [43]. Remarkably, at 12 months, there was no difference in clinical recurrence [43]. Essentially, the RCT compared a stapled side-to-side anastomosis with a hand-sewn side-to-side anastomosis. Since wound healing is different in stapled (inverted anastomosis) versus hand-sewn (mucosa-mucosa adaptation) anastomosis, this might have biased scoring the modified Rutgeerts classification.

Results from the cohort study by Gajendran et al. comparing end-to-end hand-sewn with side-to-side anastomosis showed increased healthcare consumption and more than double the amount of healthcare costs in the side-to-side group [45]. Although the authors did not observe any difference with respect to endoscopic or clinical recurrence, patients with side-to-side anastomoses had significantly lower quality of life with more abdominal complaints requiring further investigation.

Coffey’s results contradict the observation that the mesentery and bowel heals after stricturoplasty [46]. An interesting point of discussion requires further research to determine whether or not the mesentery should be resected.

## Colonic Crohn’s disease

Colonic Crohn’s disease has many different surgical treatment options including segmental resection and (sub)total colectomy, depending on disease location, severity and emergency.

### Emergency (sub)total colectomy

Therapy refractory acute colitis and imminent or actual perforation are reasons for emergency colectomy [47]. Emergency or urgent surgery for severe or fulminant Crohn’s disease, including toxic megacolon, perforation or severe haemorrhage, consists mostly of a (sub)total colectomy with construction of an end ileostomy and Hartmann closure of the rectum intraperitoneally [48]. Timing of surgery requires careful evaluation as conservative, medical treatment may save (part of) the colon, but might at the same time cause delay of surgery with increased risk of complications. This is especially important as emergency surgery in acute colitis is associated with morbidity in up to 51% of patients, including wound infection or dehiscence, intra-abdominal abscesses, small bowel obstruction, ileostomy-related complications and haemorrhage [48]. Fortunately, mortality rates in emergency surgery have decreased over the past decades, but are still around 5–10% [49–51].

### Elective (sub)total colectomy or segmental resection

The most important indication for elective colectomy in colonic Crohn’s disease is medically refractory disease. Patients with segmental colonic Crohn’s disease can be treated with both segmental resection and subtotal colectomy which are equally effective with comparable recurrence, postoperative complication and permanent stoma rates [52]. Although, according to Andersson et al. [53], segmental resection is superior to subtotal colectomy in terms of functional results with fewer loose stools and better anorectal function. Strictures in the colon are rarely managed by stricturoplasty. Apart from the technical difficulty and the little need of sparing bowel, there is a significant cancer risk in colonic Crohn’s when strictured.

When the total colon is affected by Crohn’s disease, a proctocolectomy followed by an ileal (pouch-)anal anastomosis is a possibility in the absence of perianal or small bowel disease. Unfortunately, this constitutes a select group of the Crohn’s pancolitis patients. Alternative surgical options comprise defunctioning ileostomy hoping that the proctocolon will cool down and future drugs can cure the bowel, or extensive resectional therapy. Mostly, subtotal colectomy or total panproctocolectomy with ileostomy is necessary.

### Proctocolectomy or proctectomy

Therapy refractory proctocolitis or proctitis require proctocolectomy or proctectomy and can for example be performed intersphincterically applying the transanal minimally invasive surgery (TAMIS) technique in combination with laparoscopy [54]. TAMIS is a relatively new technique using a transanal port which improves visibility and ensures safer and greater access to narrow pelvic anatomy and endangered structures compared to the conventional abdominal approach. Pelvic visualisation during proctectomy can be extra challenging due to distorted anatomy after pelvic sepsis, adhesions and fibrosis and the improved visualisation during TAMIS can then be very helpful.

As stated, rarely a restorative proctocolectomy with pouch is possible in Crohn’s disease. Ileal pouch-anal anastomosis usually results in excellent functional outcomes; however, complications including chronic sinuses, strictures, pouchitis, Crohn’s disease in the pouch and cuffitis do occur and can lead to pouch failure. An increased incidence of pouch failure is reported particularly due to wrongful indications for proctocolectomy (e.g. suspected ulcerative colitis) and reclassification to Crohn’s disease [55].

In patients with pouch failure, several different techniques for the different types of pouch failure including pouch remodelling or revision, a redo-pouch and cuff or pouch excision with permanent stomata can be performed [56]. However, this does not apply for patients with Crohn’s disease of the pouch.

In patients with only left-sided diseased colon, a hemicolectomy or hemiproctocolectomy with colo-anal anastomosis can be performed. A rectosigmoid resection can be performed using the top-down approach with laparotomy, laparoscopy or via TAMIS. TAMIS can be used to remove an inflamed, scarred rectum and can help preserve the anal sphincteric mechanism. It provides for a possible fully transanal approach but can also be combined with a transabdominal approach (laparoscopic or open).

### Surgical approach

The surgical approach in IBD must in general be minimal invasive, because it has shown to be associated with earlier recovery, less complications, fewer adhesions and incisional hernias, and preserved body image and fertility. Midline laparotomy is sometimes still necessary in patients after prior open surgery if extensive adhesions preclude a laparoscopic approach. Resection should be bowel sparing when possible, only macroscopic diseased tissue should be resected and ‘radicalism’ should not be pursued. Also, vascularisation can often be spared. All patients should be managed perioperatively in an enhanced recovery after surgery program.

## Surgical dilemma’s large bowel Crohn’s disease

### TME or close rectum resection

In patients with severe refractory Crohn’s disease, a non-restorative proctectomy can be performed either via close rectal dissection, leaving the mesorectum in situ, or via total mesorectal excision (TME). Up to now, only one study evaluated this in Crohn’s patients and showed significantly more perineal complications after close rectal dissection, and lower healing rates compared to TME [57]. This can be explained by the enhanced presence of TNF-α-producing CD14+ macrophages and reduced expression of CD206, a wound-healing marker, found in the mesorectal tissue [57]. In patients with persistent perianal wounds after close rectal excision who underwent completion total mesenteric excision with omentoplasty, clear cellular infiltrates with high expression of TNF-α mRNA were seen in the mesorectal tissue, and complete perineal healing occurred in 75% [57].

### Proctectomy or proctocolectomy

Therapeutic refractory isolated proctitis mostly in combination with severe perianal Crohn’s disease require resectional surgery. Strictly speaking, only a proctectomy is required. In a retrospective cohort from Leuven, an intersphincteric proctectomy with colostomy was performed and 90% showed severe and early endoscopic recurrence in the proximal colon after a median of 10 months, followed by completion colectomy in 50% [58]. They therefore suggest to extract the entire colon right away when the sigmoid is also affected, because within a few years after the intersphincteric proctectomy, they suspect that the colon higher up will also be affected. This concept is in contrast to the observations of the Cleveland clinic indicating completion colectomy in 5% of patients after median follow-up of 18 months, even though some of these patients had a mild proctitis prior to colectomy unlike the cohort from Leuven.

## Perianal Crohn’s disease

Perianal Crohn’s disease is present in approximately one-third of patients at time of diagnosis and the cumulative probability of any perianal disease is up to 42% within 20 years after diagnosis [59]. This disease manifestation is associated with significant morbidity and a decreased quality of life. Perianal fistulas are the most common perianal disease, but also fissures, skin tags, strictures, ulcerations, haemorrhoids and, rarely, malignancy can occur. Treatment depends on the clinical impact, which can vary significantly from asymptomatic to deeply impacting patients’ quality of life, patient preference and fistula aetiology. Fissures, skin tags and haemorrhoids often only require conservative treatment, whereas perianal fistulas may require more drastic medical and/or surgical treatment.

### Fistula location

**Fig. 5:**
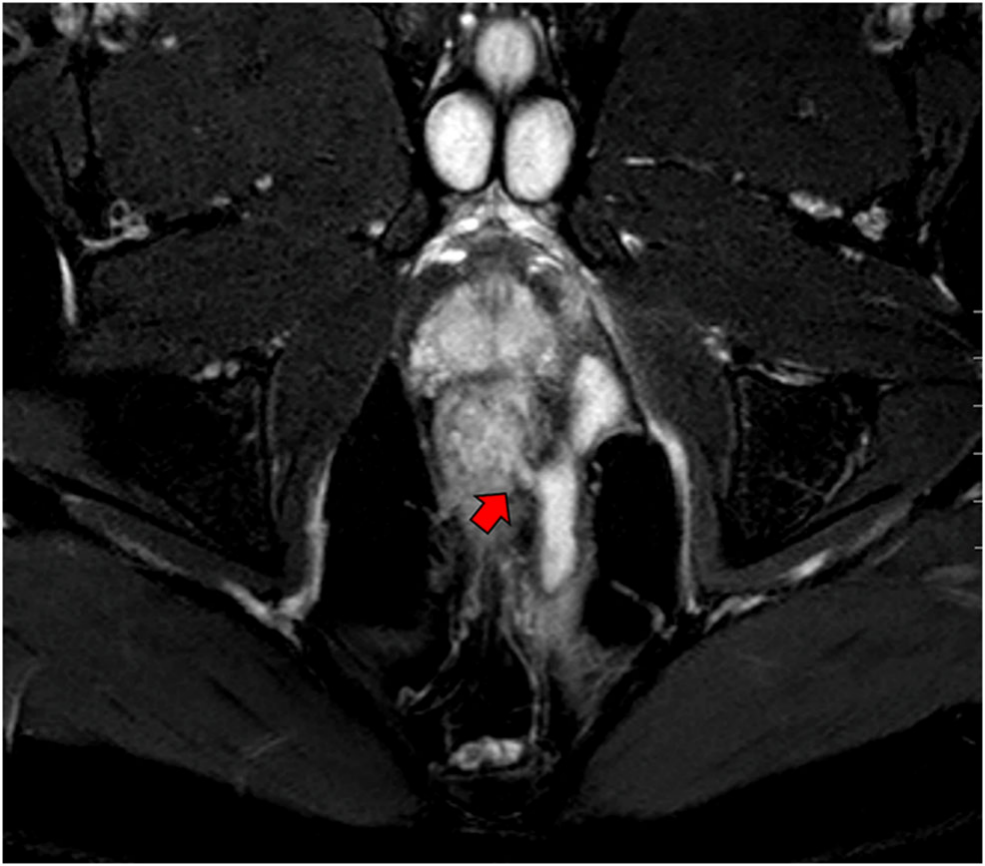
Complex perianal fistula with clear internal orfice (arrow). Coronal T2-weighted post-contrast MRI

Careful assessment with MRI (Fig. 5), colonoscopy and examination under anaesthesia is important to classify the patient for the most optimal treatment. In case of proctitis, there are no surgical options other than drainage of the sepsis and seton placements. In the absence of proctitis, the surgical options depend on the extent of tracks, and internal and external openings. Single tract and double tract with one internal opening are surgical candidates in the absence of proctitis and sepsis [60]. Perianal fistulas crossing less than one-third of the external anal sphincter can be easily treated with a fistulotomy whereas fistulas crossing more than one-third require more careful surgery. Anteriorly located tracts particularly in woman cannot be layed open because of risk of key hole incontinence. In the high fistula, fistulas are first drained by the insertion of a non-cutting seton to prevent recurrent abscess formation. A new knotless SuperSeton was recently developed, which decreases discharge, pain and cleaning problems compared to the standard knotted seton [61].

### Anti-TNF and surgical closure

The PISA trial compared chronic seton drainage to anti-TNF for 1 year and to surgical closure with a short course anti-TNF in patients with high perianal Crohn’s fistulas with a single internal opening. The authors concluded that chronic seton drainage resulted in significantly more re-interventions than anti-TNF therapy or surgical closure in a randomised setting [62]. As a consequence, nowadays, patients are counselled for anti-TNF or surgical closure of their high perianal fistula. Surgical closure can be achieved with different techniques depending on the fistula aetiology based on MRI and examination under anaesthetics. These procedures include mainly the ligation of the intersphincteric fistula tract (LIFT) procedure and the advancement flap. These procedures are not always interchangeable and depend on the height and the number of the internal fistula opening(s), and the length and shape of the fistula tract. For example, the advancement flap procedure where a flap of (sub)mucosal tissue is pulled down over the internal fistula opening can only be performed in patients where the internal opening is not too low; otherwise, the procedure could result in a ‘wet anus’ [63]. And the LIFT procedure, where the intersphincteric plane is opened and the fistula tract ligated close to sphincter, cannot be performed in patients with a u-shaped fistula with a high mid-tract section, since this has too much risk of damaging the sphincter [64]. More research on surgical closure of perianal fistulas in Crohn’s disease patients should be initiated as most are only focussed on cryptoglandular fistulas [65].

### Extensive perianal disease

Patients with extensive perianal Crohn’s disease with multiple tracts and proctitis will most likely end up with a defunctioning colostomy or proctectomy. Schlegel et al. [66] described a small series of motivated patients with proctitis and severe perianal disease who had rectosigmoid resection with coloanal anastomosis with favourable outcome in half of them.

## Surgical dilemma’s perianal Crohn’s disease

### Clinical or radiological closure as target of therapy

The aforementioned PISA trial which randomised chronic seton drainage and anti-TNF with or without surgical closure showed that anti-TNF alone is able to close the fistula clinically in less than half of the patients. Unfortunately, the large majority of these fistulas remain patent radiologically. Radiological closure was only achieved in patients that had surgical closure in combination with anti-TNF [62]. It has to be established what is most important for the patient, radiological or clinical closure. As long as the fistula is closed externally, the patient is happy. The down side is that fistula can re-open when anti-TNF trough levels are insufficient, and anti-TNF cannot be stopped [67, 68].

### Radical versus conservative surgery

The availability of a whole range of biologicals might fuel more radical surgery taking the irreversibly diseased segment out followed by more effective prophylactic therapy to avoid recurrence. In patients with a healthy colon, but severely diseased rectum and perineum, a low anterior resection under a shield of biological therapy might preserve the sphincter in these young patients. The transanal technique enables proper identification of the fistula tracts enabling adequate curettage and proctectomy. As referred earlier, Schlegel published a small series with 46% success after rectosigmoid resection with coloanal anastomosis [66].

This policy is also supported by the observations from the Cleveland clinic indicating that the proximal colon does not exacerbation after rectosigmoid resection. When the disease is limited to the rectosigmoid and the proximal colon is viable, one can opt for a rectosigmoid resection according to the total mesorectal excision principle with a low coloanal anastomosis for proctitis and perianal fistulas in a Crohn’s patient.

## Discussion

The present review shows that surgical management of the many different facets of Crohn’s disease remains challenging. Surgical management should be based on individual disease characteristics including disease location and severity. Both indication and timing of surgery require discussion in a multidisciplinary team consisting at least of gastroenterologists and surgeons. There are still some pending questions regarding timing of surgery and type of surgery.

Several surgical dilemmas remain uncertain, including what to do with the mesentery during ileocolonic resection and proctectomy, although recent evidence seems to support mesenterectomy in the latter. Also, whether a non-restorative proctectomy should be performed via close rectal dissection, leaving the mesorectum in situ, or via TME as described above, remains a point of discussion. This is dependent on the continuing discussion about the role of the mesentery in Crohn’s disease for which so far conflicting evidence is available. Other surgical dilemma’s including the role of the Kono-S anastomosis as a preventive measure for recurrent Crohn’s disease and the importance of (non)conventional stricturoplasties require further research. Future research must also help provide an answer to whether a colectomy should be performed in therapy refractory patients with proctitis when the sigmoid is also affected and whether clinical or radiological closure should be used as target of perianal fistula therapy, although in the long run the latter appears to be the case.

The role of surgery in Crohn’s disease is increasingly important. This increased importance of surgery is highlighted by both the LIR!C and PISA trials [23, 24, 62]. In the LIR!C trial, only patients with uncomplicated, non-stricturing, ileocaecal Crohn’s disease in whom conventional therapy had failed were randomly allocated to either receive laparoscopic ileocaecal resection or infliximab. Patients with clinically significant strictures or complicated disease were refused since they definitely required surgery. The study showed that laparoscopic ileocaecal resection could be used as a valid option in Crohn’s terminal ileitis being more cost effective, and can help provide a fresh start for the gastroenterologist [23, 24]. And as mentioned before, the PISA trial showed that radiological closure was only achieved in patients after surgical closure in combination with anti-TNF, not after anti-TNF alone [62].

This highlights an important issue while treating Crohn’s patients, namely the goal of treatment. Since Crohn is a benign disease survival is not one of the main goals, but rather clinical, radiological or endoscopic healing, or quality of life. This is especially important in patients with perianal fistulas since true fistula closure is still a point of discussion. Clinical healing is often considered most important since a closed external fistula opening is associated with less symptoms and an increased quality of life. However, patients often need chronic medical therapy and once they fall below the trough level, the fistula reopens or the patient has a recurrent abscess. Radiological perianal fistula closure shows prognostic value in predicting clinical recurrence and could be used when the treating physician considers stopping postoperative anti-TNF treatment [69]. These two goals, clinical or radiological, must be weighed against each other, keeping in mind what is most important to the patient and, therefore, shared decision-making should be applied here.

Gastroenterologists often expect medical therapy and especially biologicals to decrease the amount of surgery, but in some patients that is only true in the short term. Many patients who initially respond well to medical therapy require surgery after a few years later, and these numbers only increase in medical refractory patients. Timing of surgery is especially important, since postponing surgery will potentially make these patients suffer from their chronic problems compromising their socioeconomic life and have an increased use of expensive medical therapy, without the intended result. Delayed surgery is not always good and is associated with more complex disease and larger bowel resection, especially in patients whom had multiple cycles of biologicals over a longer period of time, with more complications and more stomata’s [35]. Patients with Crohn’s colitis with incomplete response being treated with multiple cycles of different biologicals might escape colectomy are at risk of developing colonic cancer on the long run [70, 71]. It has to be noted that inflamed colons are difficult to surveil, and if cancer has developed, it might grow rapidly in a patient on a biological [72].

Once it has been decided that surgery is the next step in treatment, another problem arises, which is the waiting time for surgery. Oncological surgery is currently prioritised over benign surgery resulting in a longer waiting time for both active and inactive (e.g. pouch surgery after subtotal colectomy) Crohn’s disease surgery [73]. A recent cohort study showed that while awaiting surgery, 13% of inflammatory bowel disease patients had to undergo acute- or semi-acute surgery, and 19% of patients with active disease and 15% of patients with inactive disease had complications while on the waiting list [73]. Also, 44% needed additional care including extra out-patient appointments in the clinic, visits to the emergency department or hospital admission [73]. Therefore, reducing the waiting time to an acceptable period could not only prevent more complex disease but also help mitigate healthcare consumption and the patients during the waiting period.

In conclusion, surgery should not be considered anymore as a last resort therapy for medically refractory or complex Crohn’s disease, but proven as a good alternative in terms of effectiveness, quality of life and costs as first-line therapy or as part of combination therapy with biologicals for certain conditions.

## Data Availability

Not applicable/publicly available
